# Hormonal induction of spawning in 4 species of frogs by coinjection with a gonadotropin-releasing hormone agonist and a dopamine antagonist

**DOI:** 10.1186/1477-7827-8-36

**Published:** 2010-04-16

**Authors:** Vance L Trudeau, Gustavo M Somoza, Guillermo S Natale, Bruce Pauli, Jacqui Wignall, Paula Jackman, Ken Doe, Fredrick W Schueler

**Affiliations:** 1 Centre for Advanced Research in Environmental Genomics, Department of Biology, University of Ottawa, Ontario K1N 6N5, Canada; 2 Instituto de Investigaciones Biotecnológicas-Instituto Tecnológico de Chascomús (CONICET-UNSAM). Chascomús. Provincia de Buenos Aires, Argentina; 3 CIMA, Facultad de Ciencias Exactas, Universidad Nacional La Plata, 47 y 115 (1900), La Plata, Provincia de Buenos Aires, Argentina; 4 National Wildlife Research Centre, Environment Canada, 1125 Colonel By Drive, Ottawa, Ontario, K1A H3O, Canada; 5 Environment Canada, Environmental Science Centre, PO Box 23005, Moncton, New Brunswick, E1A 6S8, Canada; 6 Bishops Mills Natural History Centre, 30 Main Street, Bishops Mills, Ontario, K0G 1T0, Canada

## Abstract

**Background:**

It is well known that many anurans do not reproduce easily in captivity. Some methods are based on administration of mammalian hormones such as human chorionic gonadotropin, which are not effective in many frogs. There is a need for simple, cost-effective alternative techniques to induce spawning.

**Methods:**

Our new method is based on the injection of a combination of a gonadotropin-releasing hormone (GnRH) agonist and a dopamine antagonist. We have named this formulation AMPHIPLEX, which is derived from the combination of the words amphibian and amplexus. This name refers to the specific reproductive behavior of frogs when the male mounts and clasps the female to induce ovulation and to fertilize the eggs as they are laid.

**Results:**

We describe the use of the method and demonstrate its applicability for captive breeding in 3 different anuran families. We tested several combinations of GnRH agonists with dopamine antagonists using Lithobates pipiens. The combination of des-Gly^10^, D-Ala^6^, Pro-LHRH (0.4 microrams/g body weight) and metoclopramide (10 micrograms/g BWt. MET) was most effective. It was used in-season, after short-term captivity and in frogs artificially hibernated under laboratory conditions. The AMPHIPLEX method was also effective in 3 Argentinian frogs, *Ceratophrys ornata*, *Ceratophrys cranwelli* and *Odontophrynus americanus*.

**Conclusion:**

Our approach offers some advantages over other hormonally-based techniques. Both sexes are injected only once and at the same time, reducing handling stress. AMPHIPLEX is a new reproductive management tool for captive breeding in Anura.

## Background

The crisis of amphibian loss and the potential detrimental ecological impacts are globally recognized [1-7]. Nace and colleagues [8,9] were probably the first to indicate the need for captive breeding and domestication of amphibians. Currently, numerous captive breeding programs directly address the problem of the amphibian decline but significant challenges still remain [10,11]. It is well known that many anurans do not reproduce easily in captivity. They either suffer from confinement stress or critical environmental cues are missing in captivity, resulting in the inhibition of reproduction [12]. It is also important to note that our basic knowledge on the reproductive biology of many amphibians, especially neotropicals, is still lacking. For some species, it is possible to simulate natural climatic conditions in captivity to induce breeding but for others hormonal treatments are required. To date there have been two main approaches for the hormonal induction of spawning, but responses are quite variable [12,13].

In vertebrates, natural ovulation in females and sperm release in males results from surge release of luteinizing hormone (LH) from the anterior pituitary gland [14,15]. One method of spawning induction in amphibians requires multiple injections of the LH-like hormone human chorionic gonadotropin (hCG). It is well known that this works reasonably well for laboratory-reared species such as *Xenopus laevis *and *Silurana tropicalis *[16]. While hCG has been tried in several other anuran species, the results range from highly variable to no success [17-19]. Another approach is to inject a brain decapeptide, gonadotropin-releasing hormone (GnRH), which is the main endogenous stimulator of LH. GnRH specifically activates GnRH receptors on LH-producing cells in the pituitary and stimulates endogenous LH production and release, which in turn acts on the gonads to increase sex steroid production and spawning. Numerous reports on the use of natural forms of GnRH and synthetic GnRH agonists exist for frogs, but results are also highly variable, and often spawning is not induced [12]. There are several reasons for this lack of success. The different GnRH forms do not necessarily maximally stimulate the pituitary to release LH. Hormone treatments will fail if either the female or the male are not in spawning condition. Hormone-based methods for captive breeding are not designed to induce sexual maturation. Rather, they are designed to induce timed release of egg and sperm so that fertilization and tadpole survival can be optimized. In this context, it is important to note that some of the protocols used to obtain reproduction in captivity involve sacrificing the parental couple, using the gametes for *in vitro *fertilization [12], which in most cases is unacceptable and must be avoided. In this context it is important to note that most of the toxicological studies using Latin American frog species still involve sacrifice of the parental couple [20-22].

In contrast to the situation for many anurans, spawning induction in some teleost fish, especially cyprinids and salmonids, has proven successful. Initially, results in fish were highly variable for much of the same reasons as described above for frogs. However, basic neuroendocrine studies revealed the presence of an important LH-inhibitory system in goldfish [23,24]. It was latter found that the catecholamine dopamine (DA) is the single most active inhibitor of basal and GnRH-stimulated LH release [25]. This led to the development of the "Linpe" method, named after the inventors H.R. Lin and R.E. Peter. Later, a fish spawning induction kit called "Ovaprim™" was produced and marketed by Syndel, Inc. in Vancouver, Canada. This method is based on the principle that blocking DA receptors in the brain and pituitary and stimulating GnRH receptors leads to a super-stimulation of LH, mimicking the LH surge to cause ovulation or sperm release in many but not all teleost fish [26-29].

The dopaminergic system has not been considered important in frog reproduction despite clear but limited evidence to the contrary. Experimentally-induced hypothalamic lesions stimulate GnRH and LH release and advances spawning in the hibernating frog *Rana temporaria *[30,31], indicating the existence of an LH-inhibitory system in the brain. The DA agonist bromocriptine can inhibit ovulation in *R. temporaria *in some situations [31]. Moreover, implantation of silastic pellets containing the dopamine antagonist metoclopromide induces ovulation in hibernating *R. temporaria *[32]. These data indicate that DA is potentially an important inhibitor of LH release in frogs as in numerous other vertebrate classes [33].

Our experience with the hormonal control of LH release and spawning in fish and fundamental studies on the role of GnRH and DA [15,34-37] provided the foundation for the current studies.

## Methods

Numerous synthetic and highly active forms of GnRH have been developed principally for clinical use in human and veterinary medicine, and some long-acting formulation have been used in some fish species. We considered des-Gly-His-(Bzl)-GnRH-ethylamide and des-Gly^10^, D-Ala^6^, Pro-GnRH the most promising because they have been shown to be biologically active in frogs ([12,18,38-40]. Similarly, among the numerous DA antagonists on the market, pimozide has been used successfully in fish [41] and metaclopromide is active in *R. temporaria *[32]. Preliminary experiments on a limited number of animals (*Lithobates pipiens *and *Ceratophrys ornata*, data not shown) and doses reported in the literature were used to establish the compounds and doses used.

### Experiment 1. Comparison of GnRH agonists and dopamine antagonists in L. pipiens

One hundred and fifty mature leopard frogs (*Lithobates pipiens, formerly Rana pipiens*; [42]) were collected near Bishops Mills, Ontario during their spring migration to breeding pools during the week of April 14, 2008. The animals were transported to the University of Ottawa and housed in large 380 L high-density polyethylene (Rubbermaid^®^) tanks containing 200 L of water and covered with a mesh to prevent escapes or predation by birds and racoons. In order to simulate spawning, tree branches were weighted to submerge them, and a few dried leaves were added to each tank as substrate. The frogs were provided with a floating piece of flat styrofoam so that they could leave the water and bask as required. Mean ( ± SD) body weights for females and males were respectively 43 ± 8 and 27 ± 6 g. Two mature females and 3 mature males were placed in each tank. This female to male ratio is the same at that determined during the spring migration collections. Animals were injected between 15:00-18:00 h on 21 April, 2008. There were 12 females and 18 males in each of 4 groups. The control animals were injected intraperitoneally with saline (0.7% NaCl; 4 μl/g) and DMSO (1 μl/g) vehicles using a 26 gauge needle attached to a disposable syringe. Treatment 1 (T1) consisted of des-Gly^10^, D-Ala^6^, Pro-LHRH (Bachem H4070.0005; 0.4 μg/g body weight; GnRH-A) and pimozide (Sigma P1793; 10 μg/g BWt, PIM). Treatment 2 (T2) consisted of GnRH-A; 0.4 μg/g BWt) and metoclopramide (Sigma;10 μg/g BWt. MET). Treatment 3 (T3) consisted of des-Gly-His(Bzl)-LHRH-ethyamide (Sigma L-2761; 0.4 μg/g BWt; GnRH-B) and PIM (10 μg/g BWt). Water temperature in the tanks varied between 19-21°C at the time of injection (Day 1) and respectively 15-17°C, 15-16°C and 12-13°C on Day 2, 3 and 4. Every morning, tanks were surveyed for the number of couples in amplexus and for the presence of eggs. The spawning data from each treatment was compared to control data using Fisher's Exact test.

### Experiment 2. The combination of GnRH-A + MET induces spawning in L. pipiens after one month in outdoor tanks

Seventy-four animals were collected in during the migration in early April 2009, which was approximately 2 weeks earlier than in 2008 because spring temperatures warmed relatively early in this year. However, temperatures, then, declined to seasonal norms, which are below those optimal for breeding (6-10°C), and it was therefore necessary to keep the animals for 3 weeks in the outdoor tanks until water temperatures warmed. We considered this to be a simulation of a possible scenario representative of some field situations where it could be necessary to hold animals for a short period prior to breeding attempts. Mean ( ± SD) body weights for females and males were respectively 52 ± 10 and 25 ± 9 g. Animals were housed as in experiment 1. They were provided with a floating piece of flat styrofoam for basking but no spawning substrate was provided until the day before injections. On April 25, 2008, the control animals were injected i.p. with saline (0.7% NaCl; 4 μl/g) and DMSO (1 μl/g) vehicles. A second group was treated with 0.4 μg/g GnRH-A plus 10 μg/g MET (T2 above), which was determined to be the most effective in experiment 1. The third group (treatment 4; T4) was injected with 0.4 μg/g GnRH-A plus 5 μg/g MET to determine if a lower dose of MET was also effective. There were 10 females and 15 males in control and T2 while there were 9 females and 15 males in T4. Water temperature in the tanks varied between 15-16°C at the time of injection (Day 1, between 13:00-15:00 h) and respectively 14-16°C, 16-17°C and 14-16°C on Day 2, 3 and 4. Every morning, tanks were surveyed for the number of couples in amplexus and for the presence of eggs. The spawning data from each treatment was compared to control data using Fisher's Exact test.

### Experiment 3. Use of GnRH-A + MET for out-of-season breeding in L. pipiens

Eight females (48 ± 9 g Bwt) and 15 males (27 ± 10 g Bwt) collected near Bishops Mills, Ontario during the fall migration in late September and early October, transferred to The University of Ottawa and used in this experiment. On October 6, 2008, captured animals were treated with tetracycline in the water (100 mg/L) for one hour, rinsed and kept in 1:20 Ringer's solution for the duration of the artificial hibernation protocol (see Table 1). Briefly, animals were cooled slowly overnight from room temperature to 4°C in an environmental control chamber (Can-Trol Environmental Systems, Inc., Markham, Ontario) in darkness, and thereafter submitted to gradual changes in temperature (range 4-15°C) and photoperiod (range 0-14 hr) according to Table 1. Lighting was provided by a 100W Phillips Natural Light bulb (1200 lumens). Four to 5 animals were kept in polycarbonate cages containing 20 l of 1:20 diluted Ringer's solution which was changed bi-weekly until Nov 14, 2008. Ringer's solution consists of 0.1 M NaCl, 1.8 mM KCl, 2 mM CaCl_2_, 1 mM MgCl_2_, and 300 mg/L NaHCO_3_. In an attempt to maximize out-of-season sperm production, males were primed with 2 injections of GnRH-A (0.025 μg/g; Oct 24, and 0.05 μg/g; Nov 2). On Nov. 11, animals were moved to a rectangular green fibreglass spawning tank (360 L water; 16-17°C; 8 h light/16 h dark) in the University of Ottawa Aquatic facility with leaves and branches as in experiment 1. They were provided with a floating piece of flat styrofoam so that they could leave the water and bask as required. Frogs were also provided with a diet of mealworms that were readily eaten. On Nov. 14 (Day 1), females and males were injected with 0.4 μg/g GnRH-A plus 10 μg/g MET mixture as in Experiment 1 and 2. The 3 days between Nov. 11-14, before injections, served as a control period and no breeding activity was observed.

**Table 1:** Artificial hibernation protocol for *L. pipiens*

Date (2008)	Day	Temp (C)	Photoperiod (on-off)
Oct. 6	1	4	no light
Oct. 10	5	4	no light
Oct. 23	17	6	15:00-18:00
Oct. 24	18	6	09:00 - 1800
Oct. 29	23	8	08:00 - 19:00
Nov. 2	27	10	06:30 - 20:00
Nov. 5	30	13	06:00 - 20:00
Nov. 9	34	14	06:00 - 20:00
Nov. 10	35	15	06:00 - 20:00
Nov. 14	39	15	06:00 - 20:00

### Experiment 4. Application of AMPHIPLEX for captive breeding in Argentinian frogs

Captive breeding of difficult species in many zoos typically involves only few animals because they are rare or endangered and often little is known about their reproductive biology. Here we demonstrate the applicability of AMPHIPLEX to 3 species from Argentina.

#### *Ceratophrys ornata*

A large female (482 g) and male (187 g), originally collected in 2004 from the flood plain of "Arroyo el Pescado" (35°01'08"S; 57°51'28"W) near La Plata City in Buenos Aires province, were housed in a 1 m^2 ^terrarium containing substrate and water simulating their natural habitat at the Facultad de Ciencias Exactas of the Universidad Nacional La Plata (UNLP) for a 5 year period. They were kept at 25°C ± 1 with a photoperiod of 16L:8D. On August 26, 2009 they were injected with AMPHIPLEX at 10:00 in the morning.

#### *Ceratophrys cranwelli*

A large female (202 g) and male (117 g) originally collected in the Province of Formosa (24°20'44,5"S, 61°06'54,5"W) in December 2006 as tadpoles were raised in a laboratory (CECOAL, Corrientes province, Argentina) and moved to the UNLP in May 2008. They were kept in similar conditions as described above until August 26 when they were injected with AMPHIPLEX in the morning. The couple was placed in a terrarium as described for *C. ornata*

#### *Odontophrynus americanus*

The animals (5 females and 10 males) were collected at night in late February and early March 2009 during an evening spawning event in the vicinity of Verónica, Buenos Aires province, Argentina (35°28'34''S, 57°09'30''W). They were transported and housed for 30 days prior to experimentation in terraria at UNLP. Two trials were performed with this species. For trial 1, one female and 2 males were placed in each of 3 terraria consisting in fiberglass tanks of 0.7 m^2 ^containing earth and water simulating their natural habitat. All animals were injected with AMPHIPLEX on March 16, 2009 in the morning. For trial two, adult frogs were collected in March 2009 and kept in captivity as described for a period of approximately 4 months. One female and 2 males were placed in each of 5 terraria and injected with AMPHIPLEX on August 4, 2009. On average, females weighed 9.0 ± 1.0 g and males 8.5 ± 0.4 g.

For all the three species, once the fertilized eggs were obtained, they were incubated at 25 ± 1°C, phoperiod 16L:8D, pH 7.6-8.3, with aeration. They were reared in different aquaria at 10 individuals per liter until metamorphosis.

## Results

### Experiment 1. Comparison of GnRH agonists and dopamine antagonists in L. pipiens

The first animals were in amplexus on Day 2 and the first eggs were seen on Day 3 and, all eggs were laid by Day 4. All egg masses regardless of treatment were fertilized and led to development of healthy tadpoles. Results are reported in Table 2 and summarize breeding activity over the 4 day experimental period. In the control group, 4 of the 12 pairs were observed in amplexus but spawning was not observed. In treatment 1, 8 pairs were observed in amplexus and 6 spawned. In treatment 2, 12 pairs were observed in amplexus and 12 spawned. In treatment 3, 5 pairs were observed in amplexus and 6 spawned. Overall, T2 was the most effective.

**Table 2:** The effects of co-treatments with GnRH agonists and dopamine antagonists on spawning induction in *L. pipiens*

Spawning in *L. pipiens*	Egg masses (%)	p-value
**Experiment 1 (2008)**		
Control	0/12 (0)	
T1 (GnRH-A+PIM-10)	6/12 (50)	.03
T2 (GnRH-A+MET-10)	12/12 (100)	.002
T3 (GnRH-B+MET-10)	5/12 (42)	.05
**Experiment 2 (2009)**		
Control	0/10 (0)	
T2 (GnRH-A+MET-10)	6/10 (60)	.04
T4 (GnRH-A+MET-5)	4/9 (44)	.08

### Experiment 2. Spawning induction in L. pipiens kept after one month in outdoor tanks

Results are shown in Table 2. Only one pair in the control group was observed in amplexus the day following injections, however, they did not spawn. In the T2 group, 3 pairs were in amplexus on Day 2. On Day 3, 3 pairs were in amplexus and 3 egg masses were found. By Day 4, 6 of the 10 females had laid eggs. All 6 egg masses were fertilized and resulted in thousands of tadpoles. In the T4 group, 4 pairs were in amplexus on Day 2. On day 3, only 2 pairs were in amplexus when observed in the morning. However, by day 4, 4/9 females in T4 has laid egg masses. All 4 egg masses were fertilized and again resulted in thousands of tadpoles being obtained. Based on results of the Fisher's Exact test, T2 was more effective than T4.

### Experiment 3. Out-of-season breeding in L. pipiens

Two pairs were in amplexus 1 day post-injection. On the second day, 5 pairs were in amplexus and one fertile egg mass was laid. On the third day post-injection a second fertile egg mass was laid and only one pair was in amplexus. No other eggs masses were laid. The visual inspection of the eggs indicated that they started to hatch 5 days post-injection.

It was important to evaluate the developmental health of the resulting tadpoles. On December 2, 2008, a subset of tadpoles was sent to the Atlantic Laboratory for Environmental Testing at the Environment Canada facility in Moncton, New Brunswick. This is a facility with established methods for rearing *L. pipiens *tadpoles to adulthood. Tadpoles were placed in large tanks (150 liters) containing dechlorinated municipal water with standard aeration filtres. The water was renewed once a week. Tadpoles were fed daily with boiled kale and a commercial dry tadpole food. Once a week tadpoles were also fed dried algal pellets. Plastic plants and a small platform suspended at the water surface are added to the tank to prevent drowning of newly metamorphosing frogs. Once the front limbs emerge, the metamorphs (Gosner stage 42) were transferred to mixed habitat tanks Upon tail rebsorption the frogs are fed wingless fruit flies and small crickets, earthworms or meal worms as they grow. With this protocol, we raised 20 metamorphs that are now approximately 12 months post-spawning.

Other tadpoles (n = 32) were raised individually from stage 25 (weight range from 0.1-0.4 g) 8 L glass aquariums containing approximately 6 L of water. Water changes occured three times a week and feeding was boiled kale and dried tadpole food three times a week. Over the course of a 2 month monitoring period, there was 6% mortality and it took a mean of 49 ± 6 (SD) days for the tadpoles to reach stage 46 under these conditions. All were within the expected normal ranges. Mean ± (SD) SVL was 26 ± 1.8 mm and mean ± (SD) wet weight was 1.8 ± 0.3 g. These values were similar to stage 46 animals originating from in-season naturally spawned eggs (~24 mm SVL and ~1.5 g) we have raised previously under similar conditions.

### Experiment 4. Captive breeding in Argentinian frogs

#### Ceratophrys ornata

The male *C. ornata *began to sing 3.5 h after injection of AMPHIPLEX. Six hours later the pair was in amplexus and the day after approximately 2000 eggs were found individually and dispersed all around the container and stuck to vegetation and other substrates like the aquarium walls and stones. Approximately 1500 eggs hatched, resulting in healthy tadpoles. The eggs reached Gosner stage 25 by 72 h after fertilization. At day 17 they were at stage 37 and the first metamorphosis were seen at day 28.

#### Ceratophrys cranwelli

The male *C. cranwelli *began to sing 15 min after injection. The pair was in amplexus 4 hours later. The following morning approximately 900 eggs were found and 870 tadpoles were obtained from these fertilized eggs. They reached Gosner stage 25 by 96 h after fertilization, reaching stage 37 at 24 days and the first metamorphosis were observed by day 41.

#### Odontophrynus americanus

In this species, some males started to sing 20 minutes following injection, and females laid eggs the following day. In trial 1, all 3 females produced fertilized eggs. In trial two, in the middle of the Argentinian winter 3/5 pairs spawned, producing approximately 3000 fertile eggs in total and 2300 healthy tadpoles were obtained. The animals in the other terraria did not spawn. Fertilized eggs developed normally reaching stage 25 at day 6, stage 37 at day 48 and the first metamorphosis observed by 76 days.

## Discussion

We present a novel method to successfully induce spawning for 1 North American and 3 South American anurans having different reproductive strategies. It is well known that ovulation and sperm release in vertebrates are under the control of the LH [15]. The surge release of LH is naturally induced by species-specific environmental conditions and controlled by a multitude of endogenous neuroendocrine factors and sex steroids [15,35]. However, reproduction can be inhibited by multiple stresses, including handling and captivity [43,44]. For example, confinement stress can inhibit reproduction in male rough-skinned newts *(Taricha granulosa) *[45]. The combination of the absence of key environmental cues and confinement stress are among the main causes for unsuccessful breeding of many anurans in captivity. The AMPHIPLEX method was designed to maximally stimulate endogenous LH release thereby inducing a coordinated release of gametes in both females and males, overriding the detrimental effects of stress in captivity.

The combination of a highly active GnRH agonist and a specific DA receptor antagonist offers significant advantage over other hormone-based anuran spawning induction methods. Results using hCG seem to be highly variable in amphibians. One possible explanation for this is that hCG is a human hormone and it does not stimulate the animal to release its own pituitary gonadotropic hormones. Rather, it only partially mimics the actions of anuran LH to activate gonadotropin receptors in the ovary or testis. This was indicated earlier by Licht (1995), and ranids in particular respond poorly if at all to hCG [18,46]. The AMPHIPLEX method stimulates endogenous LH release, thus avoiding some of the problems seen with heterologous gonadotropins. Most previous methods used multiple injections of a variety of GnRH agonists, sometimes in combination with hCG. This is more labour-intensive, and requires multiple handling of animals, which in itself can be highly stressful [45]. Furthermore, the administration of multiple injections of hCG may also make the animals refractory to the treatment as already demonstrated in fish [47]. By treating frogs with MET, the inhibitory actions of the catecholamine DA on LH are blocked, and GnRH-A more effectively stimulates spawning.

The research groups of R.E. Peter in Canada and H.R. Lin in China pioneered the use of DA antagonists for induction of spawning in fish. However, it was J. Sotowska-Brochocka [30-32] who first made the suggestion that DA may be an important inhibitor of LH release and ovulation in hibernating *R. temporaria*. Until the current report, this idea had been largely ignored by those attempting hormonal induction of spawning in Anura. Browne et al. [48] tested a single dose of pimozide in *Anaxyrus fowleri*. The authors concluded that its effects to improve hormonal induction in anurans were uncertain. Therefore, we are the first to use the combination of a highly active GnRH agonist and a DA antagonist to induce successful spawning in several anurans.

In *L. pipiens *caught as mature animals in spring 2008 and 2009, we achieved spawning rates (60-100%) highly comparable to those reported for cultured cyprinid fish with a similar single treatment of the combination of GnRH agonists and DA antagonists [26-28]. In frogs, similar doses of LHRH-A have been shown to induce sperm release in *Lepidobatrachus laevis *[39], while much higher doses of GnRH-A can induce ovulation in *Eleutherodactylus coqui *[18]. However in the Wyoming Toads (*Anaxyrus baxteri*), animals must first be primed with multiple injections of low levels of hCG and GnRH-A in combination to achieve 70-89% spawning success in response to relatively high ovulatory doses of hCG plus GnRH-A [40].

Our data show that both the type of GnRH agonist and DA antagonist influences reproductive outcome. The results from experiment 1 indicate that GnRH-A plus MET is better than GnRH-A plus PIM, indicating that MET is more effective. Comparison of T2 and T3 in experiment 1 suggests that GnRH-A is more effective than GnRH-B. The exact reason for this is unknown but it likely relates to a combination of higher affinity for the pituitary GnRH receptor and a lower degradation rate of GnRH-A compared to GnRH-B in *L. pipiens*. Our results on DA antagonists are in contrast to data in goldfish where PIM is more potent than MET in regards to the potentiation of LH release and ovulation induced by GnRH-A [49]. In experiment 2 in spring 2009, we repeated treatments of *L. pipiens *with GnRH-A and MET. It was shown that 10 μg/g Bwt MET in combination with 0.4 μg/g GnRH-A was somewhat more effective than 5 μg/g Bwt MET in combination with 0.4 μg/g GnRH-A. Therefore, we proceeded to test 10 μg/g Bwt MET in combination with 0.4 μg/g GnRH-A in several scenarios of captive breeding. Additionally, spawning success in *L. pipiens *was lower in 2009 than 2008. There are 2 main reasons for differences between years. In 2009, we recorded a major failure in juvenile recruitment in the wild population at Bishop's Mill, Ontario where the animals were harvested. This suggests that animals did not overwinter well in 2008-2009. Moreover, actual temperatures in the outdoor tanks at the time of spawning induction were on average 5°C lower in 2009 than in 2008. It was also reported in goldfish that a similar difference (8°C) in water temperature can dramatically affect the LH response to the combined injection of GnRH-A and a DA antagonist [41]. For example, peak induced LH levels can be 3-7 times lower at 12°C compared to 20°C for sexually mature female goldfish in the breeding season [41].

Presumably mature female and male *L. pipiens *were caught during the autumn migration when these frogs return to deeper bodies of water for hibernation. We developed an artificial hibernation protocol in which there were no deaths recorded. In experiment 3, we were able to induce out-of-season breeding in 2/8 females that resulted in thousands of viable tadpoles. The reason for this low success rate compared to the in-season breeding is currently unknown, but likely relates to the short hibernation period we induced. Perhaps a longer period of hibernation at 4°C would increase spawning success by promoting vitellogenesis in the females [50,51]. Nevertheless, the tadpoles grew and metamorphosed normally, although they resulted from spawnings in captivity that were approximately 6 months earlier that the expected time of natural spawning of *L. pipiens *in Eastern Ontario. An example of one representative animal resulting from this out-of-season breeding is shown (see Additional File 1, Figure S1).

The AMPHIPLEX method was also effective in the 3 tested species from Argentina. The single pair of large *C. ornata *tested in experiment 4 had been in captivity for 5 years without spawning naturally. This couple produced viable eggs following a single treatment. The tadpoles grew and metamorphosed quickly as expected for this species. Horned-frogs of the genus *Ceratophrys *are explosive breeders and their macrophagic carnivorous larvae develop in highly ephemeral ponds and have extremely short larval periods with rapid morphological development [52]. We also tested *C. cranwelli*, which also produced fertilized eggs. It is important to note that this was in the winter period of the Southern Hemisphere, which also represents a successful out-of-season breeding because *C. cranwelli *is a fossorial species that usually breeds during spring and summer time in the first heavy rains of the year in Formosa Province, Argentina. The method was also successful in *O. americanus*, which is another fossorial explosive breeder, but occurs in open grasslands and savannahs. During breeding time it is found at shallow, temporary ponds and flooded areas. On average, from the 2 trials with *O. americanus*, we were able to successfully induce spawning and the production of tadpoles from 6/8 (67%) females. In the three tested species from Argentina, AMPHIPLEX promoted reproduction. Males started to sing and display reproductive behaviour ending in amplexus and spawning in a similar manner that has been described for breeding in nature. We provide photographic evidence of successful spawning and development of individual representatives of 3 Argentinian species at various metamorphic stages (see Additional File 1, Figure S1).

## Conclusions and conservation issues

We developed a method for the effective induction of spawning in 4 frog species. Injection of a combination of a GnRH agonist and a DA antagonist is an advance over other approaches because only a single injection is required. Animals are handled less, it is quick and provides a new reproductive management tool for timed and captive breeding. Across the various treatments we harvested 33 fertile egg masses from *L. pipiens*. We did not count the number of eggs or tadpoles because there were many thousands. Egg production is variable in *L. pipiens *(800-7500 per female; [53]) but if one assumes a conservative average of approximately 4000 eggs per female, the in-season breeding resulted in >130,000 eggs during the 3 days of the experiment in the 2 years. It should also be noted that once the animals were selected and in the tanks, it took 2 people between 2-3 hrs to weight and inject the 120 animals in experiment 1 and the 74 animals in experiment 2. AMPHIPLEX is efficient and easy method to obtain large numbers of *L. pipiens *eggs for timed collections.

Captive breeding is a critical step for the conservation of threatened and endangered species. Captive breeding can also serve as a management tool to obtain tadpoles for basic research on developmental biology. We chose to use *L. pipiens *as our main test species because its reproductive biology and general physiology is relatively well-known and it is used extensively in ecotoxicological research. Nevertheless, it remains a very difficult species to successfully breed in captivity and is a very poor responder to hCG [17]. However, the AMPHIPLEX method proved highly effective in *L. pipiens*. Significantly, it was Nace (1968), more than 40 years ago, who recognized that some populations of *L. pipiens *in the U.S.A. were already declining in the mid-1960s. To address this problem, he and his colleagues established the Amphibian Facility at the University of Michigan [8,9]. However, this initiative failed because captive breeding of *L. pipiens *was not possible. Today, *L. pipiens *is still very common in many areas but Western North American populations have dramatically declined [54,55]. The State of New Hampshire has listed *L. pipiens *as a species of Special Concern, as there is an apparent decline in much of New England [56]. *L. pipiens*, may serve as a good model to use for the further development and refinement of spawning induction methods for endangered ranids.

We also studied some South American frogs with variable population status. *C. ornata *is found in the Pampas region of Argentina, Uruguay and Brazil, and it is in decline and is currently listed as 'Near Threatened' by IUCN [57], but may be highly vulnerable [58]. Captive breeding and release of *C. ornata *in the near future could perhaps counter over-exploitation in the pet trade and avoid major declines. *C. cranwelli *has a geographic range from the Chacoan region of Argentina, Bolivia, Brazil, and Paraguay has been listed as "Least Concern" in view of its wide distribution, tolerance of a broad range of habitats, but the population trend is nevertheless decreasing [59], and may be threatened by commercial and illegal capture [58].

Currently, *O. americanus *has a wider distribution than *C. ornata *[60]. It exists as a species complex with both diploid and tetraploid populations [61]. While listed as a species of "Least Concern" [60], Lavilla and Cei (2001) [58] consider it's status as undetermined and little is known about the various members of this complex. To date, only the tetraploid population of *O. americanus *has been found in Buenos Aires Province (Rosset, S.S., pers. comm.). Induction of spawning in this member of Cycloramphidae provides the possibility to further characterize the development and reproduction of *O. americanus *and related species.

## Competing interests

The authors declare that they have no competing interests.

## Authors' contributions

VLT developed the AMPHIPLEX method, designed, performed all experiments with *L. pipiens *and wrote the paper. GMS helped develop the AMPHIPLEX mixture and performed injections in Argentinian frogs. GN collected Argentinian frogs, housed and cared for them and their offspring. BP and JW helped design and perform the spawning experiments with *L. pipiens*. PJ and KD developed the hibernation protocol. FS collected all *L. pipiens *and contributed to development of spawning conditions required. All authors read and approved the final manuscript.

## Supplementary Material

Additional file 1 **Supplemental figure 1:>** Froglets and tadpoles resulting from induced spawning using the AMPHIPLEX method. Shown are photographs of *Lithobates pipiens, Ceratophrys ornata, Odontophrynus americanus *and *Ceratophys cranwelli*. Click here for file
