# Supplementary material for: Hormonal induction of spawning in 4 species of frogs by coinjection with a gonadotropin-releasing hormone agonist and a dopamine antagonist
Source: Reprod Biol Endocrinol. 2010 Apr 16;8:36. doi: 10.1186/1477-7827-8-36 (PMC2873446; doi:10.1186/1477-7827-8-36)
Supplement: Additional file 1 — Supplemental figure 1:> Froglets and tadpoles resulting from induced spawning using the AMPHIPLEX method. Shown are photographs of Lithobates pipiens, Ceratophrys ornata, Odontophrynus americanus and Ceratophys cranwelli. [file 1477-7827-8-36-S1.PDF]

Supplemental Figure 1: Trudeau, VL et al.

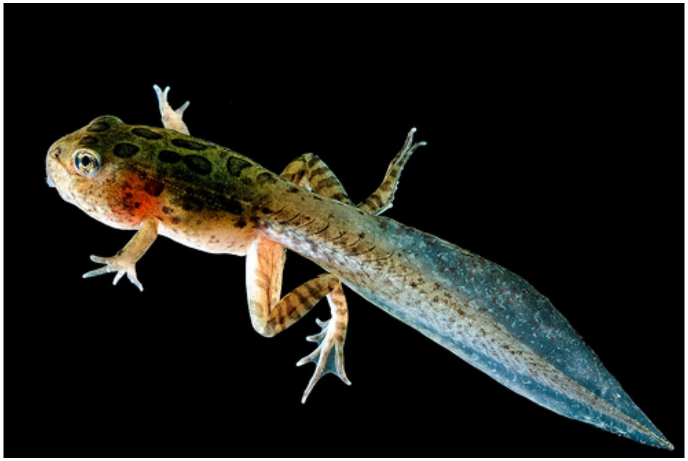

Lithobates pipiens

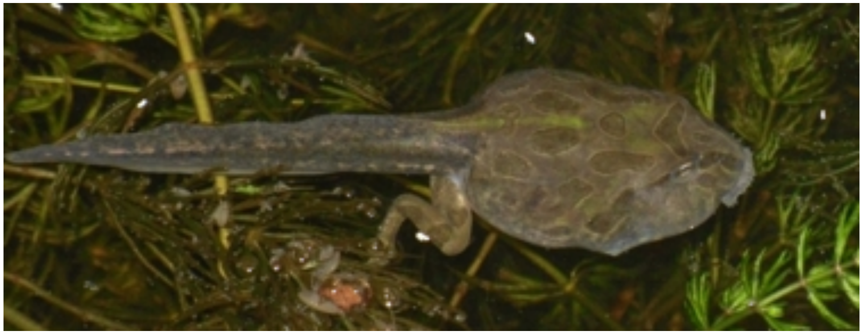

Ceratophrys ornata

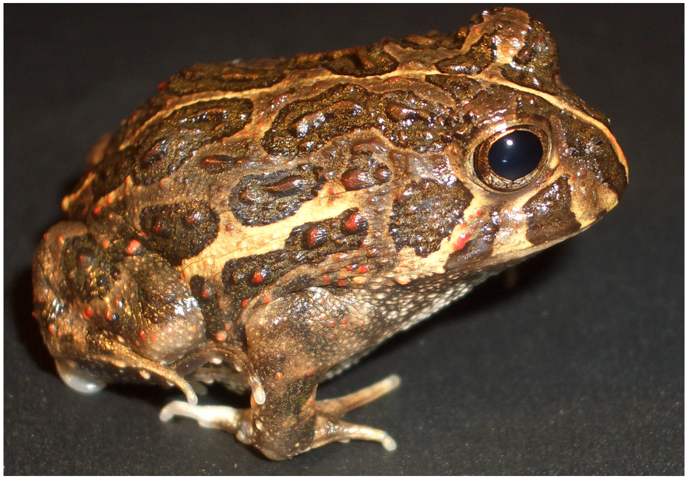

Odontophrys americanus

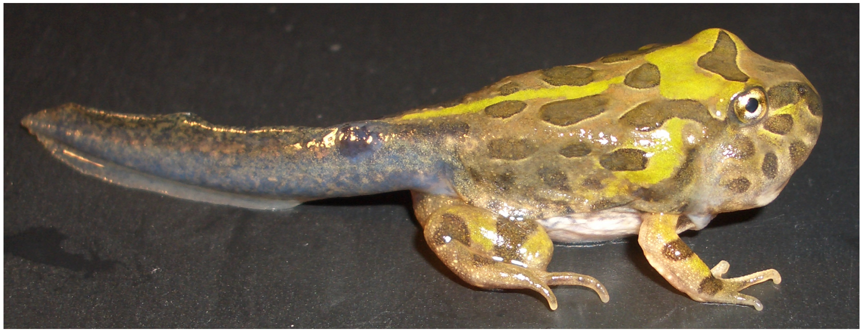

Ceratophrys cranwelli

Photo credits: A. Morin, *L. pipiens*; G. Natale, *C. ornata* and *O. americanus*; L. Alcalde, *C. cranwelli*
